# Outbreak of Uncommon O4 Non-Agglutinating *Salmonella* Typhimurium Linked to Minced Pork, Saxony-Anhalt, Germany, January to April 2013

**DOI:** 10.1371/journal.pone.0128349

**Published:** 2015-06-01

**Authors:** Katja Alt, Sandra Simon, Carina Helmeke, Claudia Kohlstock, Rita Prager, Erhard Tietze, Wolfgang Rabsch, Ioannis Karagiannis, Dirk Werber, Christina Frank, Angelika Fruth

**Affiliations:** 1 Department for Infectious Disease Epidemiology, Robert Koch-Institute, Berlin, Germany; 2 National Reference Centre for *Salmonella* and other Bacterial Enterics, Robert Koch-Institute, Wernigerode, Saxony-Anhalt, Germany; 3 State Agency for Consumer Protection of Saxony-Anhalt, Magdeburg, Saxony-Anhalt, Germany; Beijing Institute of Microbiology and Epidemiology, CHINA

## Abstract

**Introduction:**

In January 2013, the National Reference Centre for *Salmonella* (NRC) detected a salmonellosis cluster in Saxony-Anhalt, Germany, caused by uncommon O4 non-agglutinating, monophasic *Salmonella* (*S*.) Typhimurium DT193. Circulating predominant monophasic *S*. Typhimurium DT193 clones typically display resistance phenotype ASSuT. We investigated common exposures to control the outbreak, and conducted microbiological investigations to assess the strains’ phenotype.

**Methods:**

We conducted a case-control study defining cases as persons living or working in Saxony-Anhalt diagnosed with the O4 non-agglutinating strain between January and March 2013. We selected two controls contemporarily reported with norovirus infection, frequency-matched on residence and age group, per case. We interviewed regarding food consumption, especially pork and its place of purchase. We calculated odds ratios (ORs) with 95% confidence intervals (95% CI) using logistic regression. The NRC investigated human and food isolates by PCR, SDS-PAGE, MLST, PFGE, MLVA and susceptibility testing.

**Results:**

Altogether, 68 O4 non-agglutinating human isolates were confirmed between January and April 2013. Of those, 61 were assigned to the outbreak (median age 57 years, 44% female); 83% cases ≥ 60 years were hospitalized. Eating raw minced pork from butcheries within 3 days was associated with disease (31 cases, 28 controls; OR adjusted for sex: 3.6; 95% CI: 1.0-13). Phage type DT193 and MLST ST34 were assigned, and isolates’ lipopolysaccharide (LPS) matched control strains. Isolates linked to Saxony-Anhalt exhibited PFGE type 5. ASSuT- and ACSSuT phenotype proportions were 34 and 39% respectively; 54% were resistant to chloramphenicol. Three pork isolates matched the outbreak strain.

**Discussion:**

Raw minced pork was the most likely infection vehicle in this first reported outbreak caused by O4 non-agglutinating, mostly chloramphenicol-resistant *S*. Typhimurium DT193. High hospitalization proportions demand awareness on the risk of consumption of raw pork among elderly. LPS analysis indicated O4 expression; therefore, testing with antisera from different lots is recommendable in unexpected agglutination reactions.

## Introduction

On 15 February 2013, the NRC informed the Department for Infectious Disease Epidemiology at the Robert Koch-Institute (RKI) about the identification of an uncommon variant of monophasic *S*. Typhimurium DT193. Thirty-one isolates originating from stool specimens of persons with diarrhea had been collected since mid-January in Saxony-Anhalt, mainly in two municipalities. Notably, these isolates did not show the typical agglutination of *S*. Typhimurium 4,[5],12:i:- when mixed with the O4 antiserum in use for routine diagnostics (Sifin monoclonal O4 antiserum Ref. no. TR5410 and lot no. 1401011).

Salmonellosis is the second most commonly notified bacterial disease in Germany (23.2/100.000 persons in 2013) [1]. *Salmonella* (*S*.) Typhimurium has become the most frequently determined serovar and accounted for 41% of serotyped human *Salmonella* isolates in 2013 [1]. *S*. enterica serovar 4,[5],12:i:- is considered a monophasic variant of serovar Typhimurium 4,[5],12:i:1,2 [2]. The monophasic variant has been increasingly reported worldwide. It is most commonly isolated from pigs, pork and both sporadic and outbreak-associated cases of human salmonellosis with high prevalence of hospitalization [3–9]. The dominant phage type among *S*. Typhimurium 4,[5],12:i:- in German human and isolates from pigs and pork is DT193 [5]. Currently, monophasic *S*. Typhimurium strains characteristically display the resistance phenotype ASSuT (Ampicillin (A), Streptomycin (S), Sulfonamides (Su) and Tetracycline (T)) encoded by chromosomal genes *bla*_TEM-1_, *strA-strB*, *sul2* and *tet(B)* [10,11]. Additional resistance to chloramphenicol in the phenotype ACSSuT is typically found in biphasic *S*. Typhimurium DT104 strains—the most prevalent *S*. Typhimurium strain type for years in Germany before the monophasic variant rapidly spread in the first decade of the 2000s [4]. In *S*. Typhimurium DT104 the genes *bla*_PSE-1_, *floR*, *aadA2*, *sul1* and *tet(G)* encode the resistance pattern and are located on the *Salmonella* genomic island 1 (SG1) [12]. To date, chloramphenicol resistance has been uncommon in monophasic *S*. Typhimurium (10 out of 558 monophasic DT193 isolates investigated by the National Reference Centre in 2013).

Serological confirmation and molecular typing of voluntarily sent isolates is performed at the German National Reference Centre for Salmonellae and other Enterics (NRC). Smooth strains of *S*. Typhimurium (and other group B serovars) are known to display O antigens 4 and 12. In addition, some *S*. Typhimurium strains also express O antigens 1 and 5, but none of these is exclusive to *S*. Typhimurium [13]. These O antigens are present on the surface of bacteria and are conveniently detected by slide agglutination test. Antibodies (both polyclonal and monoclonal) directed against O antigen 4 are routinely used for the serological identification of serogroup B salmonellae. Genetically, *rfbJ* encoding the abequose synthase necessary for O4 expression, and a 1000bp fragment of the IS-element between the *fliA* and *fliB* genes are targeted to identify *S*. Typhimurium [2,14].

We investigated this outbreak in cooperation with the State Agency for Consumer Protection of Saxony-Anhalt to identify the transmission vehicle and prevent further cases. Furthermore, we characterized this new strain pheno- and genotypically to assess the uncommon phenotype. With this multidisciplinary investigation we detected the most likely vehicle of infection and provide a detailed microbiological description of this atypical strain.

## Methods

### Epidemiological investigations

#### Case definition

A case in this outbreak was defined as a German resident whose culture results yielded the outbreak strain since the beginning of December 2012 until April 2013. The outbreak strain was defined as monophasic *S*. Typhimurium phagetype DT193 lacking typical agglutination of the O4 antigen, O12 antigen positive and O5 antigen negative agglutination reactions (defined as 12:i:-), and displaying PFGE type 5 (and its clonal variants differing in one band from type 5) if typed by PFGE. Probable cases were defined as persons with gastrointestinal symptoms epidemiologically linked to cases. Probable cases were only included in description of disease clusters associated with social events.

#### Hypothesis generating and case-control study

We performed initial hypothesis-generating patient interviews using a generic trawling questionnaire for *Salmonella* outbreaks designed by the RKI. Further information collection included interviewing cases who had been at a social event attended by further probable cases. A frequency-matched case-control study started on 27 February including cases linked to Saxony-Anhalt by residence or workplace diagnosed between January and March 2013. We selected two controls for each case, frequency-matched on residence county and age group from the national reportable disease surveillance system as persons with a reported Norovirus infection between January and March 2013. This approach was chosen assuming easy access to contact data and willingness of controls to cooperate with local health authorities. Controls that were part of an infection cluster or institutionalized were excluded from analysis. Recruitment of cases and controls was performed by the local health authorities. Telephone interviews were conducted by trained persons using an outbreak-specific questionnaire. Based on the results of hypothesis-generating interviews we collected data on the consumption of raw and cooked minced pork, eggs, yoghurt, hard cheese, chocolate bars, cake and tea for the week before onset of symptoms. Information on minced pork included purchase addresses and whether it had been bought at the butchery or prepackaged from supermarket cold stores, and whether it had been eaten within 3 days or more before the onset of symptoms. We also collected data on demographics, treatment with antacids and contact with ornamental birds.

This study did not require separate ethical approval since outbreaks are routinely investigated according to German Infection Protection Act. For the effective termination of the outbreak all participants were asked to give oral consent before being interviewed on the telephone. Consent was recorded in a separate form. Interviews on cases younger than 18 years were conducted with parents instead of minors. Data were analyzed anonymously.

### Statistical analysis

We entered data from questionnaires into an Excel spreadsheet. We calculated proportion of hospitalizations. We performed single variable and multivariable logistic regression to calculate odds ratios (ORs) and 95% confidence intervals (95%CI) for the strengths of the associations between exposure and disease using STATA 12 (StataCorp, TX). We tested interactions, and adjusted for potential confounders by inclusion in the final model. Significance level was set at p<0.05.

### Microbiological investigations

All isolates underwent serotyping according to the White-Kauffmann-Le Minor scheme [15] using Sifin (Sifin Diagnostics GmbH, Berlin, Germany) monoclonal O4 (lot no. 1401011) and O5 (lot no. 1491111) antisera, and polyclonal O12 antiserum of own production (immunization with *S*. Typhi T4 and absorbed by *S*. Typhi T2).

We performed PCR according to Lim et al. for the detection of a 663 bp fragment of the O4 antigen-associated gene *rfbJ* [14]. Amplification of the *fliA-fliB* intergenic region for the detection of a specific *S*. Typhimurium 1000 bp fragment was done according to Echeita et al. [2]. Further characterization of isolates included screening by PCR for the presence of an 18.4 kb genomic island recently found adjacent to the *thrW* tRNA locus in *S*. Typhimurium 4,[5],12:i:- DT193 strains as described by Trüpschuch et al. [8]. Presence of the *fljB* gene encoding the 2nd phase flagellar antigen was investigated by adding two more primers [14] to the PCR by Trüpschuch et al. [8], resulting in a 526 bp product when *fljB* is present. PCR reactions were carried out using a 2720 Thermal Cycler (Applied Biosystems by Life Technologies, Carlsbad, USA) and HotStarTaq Master Mix Kit from QIAGEN (QIAGEN, Hilden, Germany). PCR products were run on 1.5% agarose gels at 120 V for 90 min. The 50 bp and the 1 kb Plus DNA Ladder (GeneRuler, Thermo Scientific Inc., Waltham, USA) were used as molecular size markers.

To investigate the molecular surface of the outbreak strain we prepared the *Salmonella* lipopolysaccharide (LPS) fraction as described by Seltmann et al. [16] followed by polyacrylamide gel electrophoresis (SDS-PAGE) and silver staining. Twenty μl per LPS sample and 5 μl prestained protein ladder PageRuler (Thermo Scientific Inc., Waltham, USA) where loaded on a 4% stacking gel poured on a 12% separating gel and run in a Mini-PROTEAN Tetra Cell (BioRad, Hercules, USA) for 90 min at 40 mA under reducing conditions. We conducted silver staining using the Pierce Silver Stain Kit (Thermo Scientific Inc., Waltham, USA) according to the manufacturer’s instructions with an additional incubation of the gel in 0.7% periodic acid for 20 minutes prior to fixation. The monophasic *S*. Typhimurium isolate RKI 06–01900 and the biphasic *S*. Typhimurium laboratory strain NTCC12023 served as controls.

We performed phage typing using the extended Anderson set [17] according to the International Federation for Enteric Phage Typing (IFEPT) as previously described [18]. We carried out multilocus sequence typing (MLST) as reported by Kidgell et al. [19] and assigned sequence types according to the MLST database http://mlst.ucc.ie/mlst/dbs/Senterica.

We characterized a subset of isolates by pulsed field gel electrophoresis (PFGE) after digestion of genomic DNA with the *Xba*I restriction enzyme according to the Pulse-Net protocol [20]. Gel images were analyzed using BioNumerics v.6.5 (Applied Maths NV, Sint-Martens-Latem, Belgium). PFGE types were assigned using a NRC-internal nomenclature. We conducted multilocus variable-number tandem-repeat analysis (MLVA) on using an ABI 310 Genetic Analyzer and assigned variable number of tandem repeats (VNTR) allele number according to Larsson et al. [21].

We determined minimal inhibitory concentrations (MICs) of antibiotics by broth microdilution according to the German standard of antibiotic susceptibility testing [22]. We set clinical break-points as recommended by the German Institute for Standardization (DIN) in standard 58940–4. If DIN breakpoints were not available, breakpoints given in document M100-S20 by the Clinical Laboratory and Standards Institute (CLSI) were used [23]. We used the protocol of Lucarelli et al. to run PCRs for detection of SG1 and resistance genes *bla*_TEM_, *strA-B*, *sul2* and *tet(B)* [11]. Chloramphenicol resistance genes *catA1*, *floR*, and *cmlA1* were amplified by PCR according to Guerra et al [24].

### Food safety investigations

*Salmonella* isolates from pork specimens (meat, minced meat and raw sausage) taken as part of the outbreak investigation or within the framework of the routine food safety sampling plan in the affected municipalities of Saxony-Anhalt were serotyped at the Department of Food Safety at the State Agency for Consumer Protection in Halle an der Saale. Anti-*Salmonella* polyvalent antiserum I (lot no. 1390812), O4 (lot no. 1051010), O5 (lot no. 1491111), H i (lot no. 310410), H 2 (lot no. 940811) antisera from Sifin (Sifin Diagnostics GmbH, Berlin, Germany) were used.

Isolates characterized phenotypically in routine diagnostics as *S*. Typhimurium, *Salmonella enterica* O4 positive and O5 positive or negative or *Salmonella enterica*, which reacted group-I positive only with a polyvalent antiserum were sent to the NRC for further characterization.

Product tracing of food items tested positive for the outbreak strain and subsequent identification of food chains were carried out in close cooperation of the regional Food and Veterinary Product Safety Authorities and the superior responsible State Administration Office.

## Results

### Epidemiological investigations

Overall, 61 cases were confirmed by the NRC as part of the outbreak (see results of microbiological investigations) in the period between January and April 2013. In addition, 23 probable cases were epidemiologically linked to confirmed cases in disease clusters.

The median age of cases was 57 years (interquartile range: 19–75). Ten (16%) cases were children aged ≤ 5 years and 27 (44%) were female. Cases lived in 10 counties in 5 federal states. Fifty-six (93%) lived in Saxony-Anhalt, 49 of them in two counties. Three cases with residency in other federal states commuted daily to Saxony-Anhalt for work. Two cases were not linked to Saxony-Anhalt through residence or workplace. The proportion of hospitalized cases was 59% overall and 83% among cases ≥60 years (24/29). No deaths were reported.

Four of 7 cases interviewed in trawling interviews reported having bought minced pork at local butcheries and eaten it within 3 days before illness onset.

Three spatiotemporal disease clusters (A-C) occurring during the outbreak involving 4 confirmed and 23 probable cases were identified during interviews. Two were located in Saxony-Anhalt, one in a neighboring federal state. Interviews with cases linked clusters A and B to two independent social events offering ready-to-eat raw minced pork. In cluster C, 10 probable cases reported having bought meat products in the same butchery. One case was an asymptomatic carrier who worked at the butchery.

For the case-control study phone numbers of 31 cases and 28 controls willing to participate in the study were provided by the local health authorities and interviewed on exposures. Cases lived in Saxony-Anhalt or commuted daily for work to five municipalities in Saxony-Anhalt. The median age was 29 years and 23% were female. Controls lived in the same municipalities as cases, had a median age of 49 years and 54% were female. Eating raw minced pork from local butcheries within 3 days before symptom onset was the only exposure significantly associated with disease (OR adjusted for sex: 3.6; 95% CI: 1.0–13). Twelve (45%) cases had been exposed, compared to 4 (15%) controls. Butchery c was mentioned by exposed cases 5 times, and a further butchery (other than a-c) 3 times as the place of pork purchase (see results of food investigations).

### Microbiological investigations

Monoclonal O4 antiserum (lot no. 1401011) used by the NRC in routine serotyping detected 68 human isolates lacking agglutination (among >700 *S*. Typhimurium isolates displaying regular agglutination) between January and April 2013. Agglutination reaction with O5 antiserum (lot no. 1491111) was negative for all isolates. PCR of the *rfbJ* gene and IS-element between the *fliA* and *fliB* genes delivered 663 and 1000 bp amplicons for all isolates. Modified multiplex PCR according to Trüpschuch et al. [8] yielded two bands of 1128 and 903 bp indicative of presence of the genomic island for all isolates.

**Fig 1:**
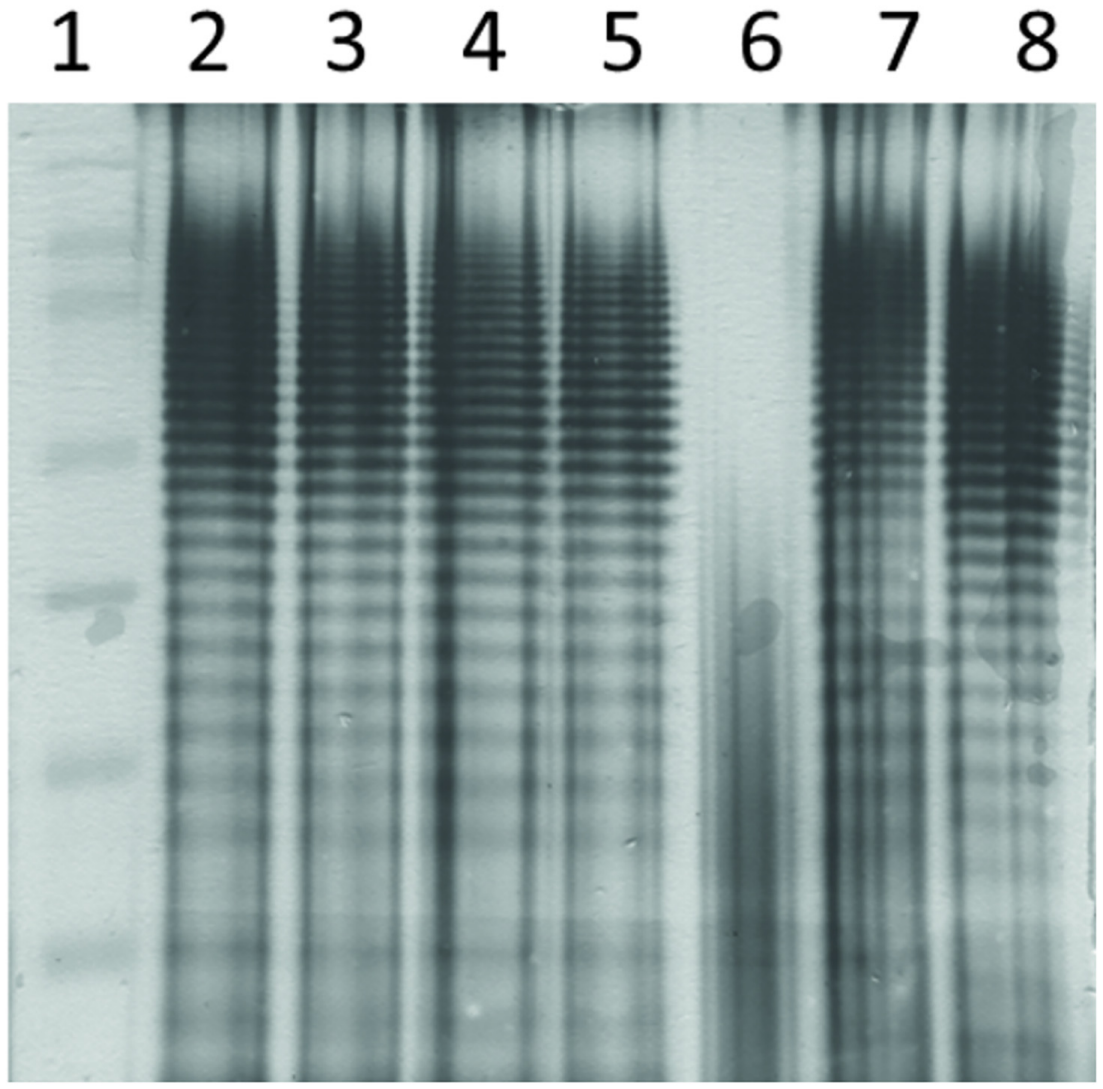
Silver-stained SDS-gel of LPS. Lanes: 1) protein ladder, 2)– 5) smooth isolates of outbreak strain, 6) rough isolate of outbreak strain, 7) biphasic *S*. Typhimurium control strain NTCC12023, 8) monophasic *S*. Typhimurium control strain 06–01900.

SDS-PAGE revealed matching patterns for smooth 12:i:- isolates and the control strains. The only rough isolate identified during the outbreak displayed a different LPS pattern (Fig 1).

Phage type DT193 was assigned to all isolates and MLST revealed ST34 for all 8 tested human isolates.

Thirty-three human isolates analyzed by PFGE exhibited PFGE-*Xba*I type 5, including its subtypes 5a and 5h with one strain each. These particular subtypes differ each in one band from type 5 and are therefore considered to be clonal variants. Table 1 shows combinations of PFGE- and MLVA patterns by patients’ residences. Of 61 human isolates assigned to the outbreak strain, 44 (72%) displayed MLVA type 3-14-10-NA-0211, and 4 single locus variants were observed among the remaining 17 (27%) strains (Table 1).

**Table 1:** PFGE and MLVA types found in 61 isolates according to patients’ residences.

	PFGE type				
MLVA type	5	5a	5h	Untyped	Total
	Federal state (isolates typed by PFGE/total isolates)				
3-**13**-10-NA-0211	ST (1/56)				1
3-14-10-NA-0211	HE (1°/1)			ST (20/56)	44
	ST (21/56)				
	SN (1/1)				
	LS (1°/1)				
3-14-**8**-NA-0211	ST (3/56)		ST (1/56)	ST (8/56)	14
	TH (2/2)				
3-14-**9**-NA-0211		ST (1/56)			1
3-**15**-10-NA-0211	ST (1/56)				1
Total	31	1	1	28	61

ST: Saxony-Anhalt, HE: Hesse, SN: Saxony, LS: Lower Saxony, TH: Thuringia. MLVA loci different from 3-14-10-NA-0211 are marked bold. °No link to Saxony-Anhalt.

Clonally distinct PFGE types 40 and 51a were found in further 7 non-agglutinating isolates from persons without link to Saxony-Anhalt.

Among 61 isolates involved in the outbreak 8 different patterns of antibiotic resistance were detected based on MICs. Proportions of ASSuT- and ACSSuT phenotypes were 34 and 39% respectively. Thirty-three (54%) were resistant to chloramphenicol according to MICs. PCR results revealed that SG1 is not present and that resistance pattern ASSuT is encoded by *bla*_TEM_, *strA-B*, *sul2* and *tet(B)*, respectively. PCRs for detection of one of the three most common genes encoding resistance to chloramphenicol in *Salmonella enterica* (*catA1*, *cmlA1* and *floR*) were negative.

### Food safety investigations

Eight isolates from pork products (4 from routine sampling and 4 taken as part of the outbreak investigation) were sent to the NRC for further characterization by the State Agency for Consumer Protection of Saxony-Anhalt. Three were non-agglutinating and displayed PFGE type 5, MLVA type 3-14-10-NA-0211, and resistance phenotype ACSSuT. MLST performed in one isolate revealed ST34. Positive food specimens were taken in 3 different butcheries (a—c). The investigation of the regional Food and Veterinary Product Safety Authorities showed that butchers a and b received pork from one supplier. This supplier was supplied by one presupplier from the federal state Lower Saxony. Butcher b was also supplied by butcher c, which processes both self-produced meat and meat from a Dutch presupplier. Investigations showed that meat contaminated with the outbreak strain was supplied by only two suppliers outside of Saxony-Anhalt. Origin stocks and slaughterhouses could not be traced. No connection was identified between butcheries a—c and disease clusters A—C.

## Discussion

This salmonellosis outbreak was caused by an uncommon phenotype of monophasic *S*. Typhimurium. It was detected at the NRC by standard routine serotyping. Based on trawling interviews we generated the hypothesis that consumption of raw minced pork was the vehicle of transmission in this large outbreak in Saxony-Anhalt in the beginning of 2013. Strong evidence for raw minced pork as the outbreak vehicle was provided by a multidisciplinary investigation, including patient interviews and a case-control study, as well as microbiological and environmental investigations involving positive food specimens.

At least 61 persons contracted disease during this outbreak. The high proportion of probable cases linked to events offering raw minced pork though, suggests higher attack rates with mild cases. The only statistically significant association with the disease in a case-control study was the consumption of raw minced pork from local butcheries within 3 days before symptom onset. Although only 45% of cases reported consumption of raw minced pork in the 3 days prior disease onset, incomplete food history recall needs to be considered given that the study started in the end of February, 50% of interviewed cases had been ill at least one month before and ~1/3 were aged >60. Ten children ≤5 years (16%) and 29 persons ≥60 years (48%) among cases indicate low compliance with current German recommendations to avoid eating raw minced pork in these vulnerable age groups.

Though controls with a Norovirus infection were picked due to their apparent ready contactability to the local health authorities in this area and season, some shortcomings of using this approach were noted. During the interviewing phase of the outbreak investigation in the beginning of March (end of the Norovirus season) fewer cases were notified than when we looked up whether there were enough to use. When interviewed, many Norovirus patients turned out to be part of clusters or institutionalized and had to be excluded which led to a smaller sample and limited statistical power. Despite the difference in median age, case and control groups were comparable on other demographics, and the controls closer in age to the cases overall. Even though the selected Norovirus patients may not be a representative sample of the population in general, we excluded those involved in outbreaks and thus have no reason to believe that they were not representative in terms of the exposures examined in the present investigation.

We addressed the question why the O4-antigen was not detectable in a total of 71 isolates (68 from humans and 3 from food) using routine diagnostic antiserum while PCR of the *rfbJ* locus encoding the abequose synthase necessary for O4 expression was positive. Since attachment of abequose to the sugar backbone of the LPS chain is essential for the assembly of the O4-antigen repeating units [25,26] a defective abequose synthesis would result in a rough phenotype of the strains. This was observed only in one of the 71 non-agglutinating strains suggesting that the biosynthesis of the O4-antigen including sugar transfer and ligation steps, and transport to the bacterial surface was generally working. The structural modification of the O4 antigen causing the failure to agglutinate with antiserum of the specific lot in use was not revealed. Apparently the epitope shows only a minor variation since the antigen could still be recognized with antiserum of other lots, and LPS shows no difference to control strains as demonstrated by silver-stained SDS-PAGE. Blocking or masking of an epitope occurs sporadically but often won´t be detected, as long as routine agglutination with most available antiserum lots still works. Such events only come to our attention when particular strains cause outbreaks and are therefore detected in greater numbers.

Although neither the O4 nor the O5 antigen could be confirmed by routine slide agglutination for *Salmonella* serotyping with the particular antiserum lot in use, other diagnostic approaches including phage typing, PFGE, MLVA and MLST revealed that isolates belonged to *S*. *enterica* serovar Typhimurium. This was additionally confirmed by the amplification of the B-group determining *rfbJ* gene, encoding the abequose synthase necessary for O4 expression, and the Typhimurium-specific location of an IS-element between the *fliA* and *fliB* genes, respectively.

Further analyses showed that the 12:i:- strains isolated from humans and pork share traits with the monophasic *S*. Typhimurium predominant strain type in Germany: isolates belonged to phage type DT193 and harbored the typical 18.4 kb genomic island at the *thrW* tRNA locus, while the *fljB* gene encoding the 2^nd^ phase flagellar antigen was not detectable by PCR. Moreover, MLST revealed ST34, a sequence type characteristic for monophasic *S*. Typhimurium [27].

PFGE results specified the extent of non-agglutinating isolates involved in the outbreak in Saxony-Anhalt. Among isolates with type 5 only 2 were not linked to patients with residence or workplace in Saxony-Anhalt, whereas isolates from patients of other federal states, also unlinked to Saxony-Anhalt, displayed other types than 5 and its variants 5a and 5h (data not shown) and can therefore be considered as from cases of sporadic salmonellosis. PFGE results conclude that 12:i:- strains do not belong to a single clone.

Resistance pattern ASSuT encoded by *bla*_TEM_, *strA-B*, *sul2* and *tet(B)*, and absence of SG1 are typical for monophasic *S*. Typhimurium. However, the exact mechanism behind heterogeneity of resistance patterns was not resolved. Since we could not detect common genes conferring resistance to chloramphenicol in *Salmonella enterica* and MICs where generally low (data not shown) we suspect an unspecific efflux pump or decreased membrane permeability rather than a specific chloramphenicol-resistance gene. This is supported by the fact that chloramphenicol-sensitive and resistant isolates shared the same PFGE types (data not shown). However, a large proportion (54%) of the 12:i:- strains seem to have developed a method to block the antimicrobial effects of chloramphenicol—a characteristic that has been uncommon so far in monophasic *S*. Typhimurium (10 out of 558 monophasic DT193 isolates typed at the NRC in 2013 [1.8%]).

Our experimental results revealed that the 12:i:- strains share fundamental characteristics with the monophasic *S*. Typhimurium DT193 strains circulating in Germany. Based on these findings we conclude that the uncommon isolates most probably originated from this particular monophasic strain type, and acquired resistance to chloramphenicol. However, the reason for the failing detectability of the O4 antigen and the mechanism of resistance could not be clarified with the applied methods. Colonies of *Salmonella* showing a smooth phenotype and of suspected serovar (based on flagellar antigens, phage type etc.) with undetectable O-antigen should be tested with antisera from lots distinct from the one in use, since masking of epitopes may occur.

Although the source of contaminated pork batches could not be traced back and our case-control study is of limited statistical power due to hampered recruitment of controls (only 28 agreed to participate) we provided epidemiological and microbiological evidence that raw minced pork was the most likely vehicle of transmission during this salmonellosis outbreak affecting at least 60 persons in Saxony-Anhalt in 2013. The high proportion of hospitalization among elderly and a high degree of ill children ≤5 during this outbreak call for more awareness and information on the risk of eating raw pork among these vulnerable populations.

## Supporting Information

S1 Table Isolate information. (XLS)
